# Developing and Implementing Postoperative Pain Management Guidelines for Breast Cancer Surgery: A Leadership Perspective

**DOI:** 10.7759/cureus.50951

**Published:** 2023-12-22

**Authors:** Mouza Al Ameri, Nandan M Shanbhag

**Affiliations:** 1 Department of Oncology, Tawam Hospital, Al Ain, ARE; 2 Department of Internal Medicine, United Arab Emirates University, Al Ain, ARE

**Keywords:** patient-centered care, heath-related quality of life, pdsa cycle, quality improvement, uae healthcare, evidence-based guidelines, healthcare leadership, patient satisfaction, postoperative pain management, breast cancer

## Abstract

Introduction

Persistent postoperative pain significantly diminishes the quality of life in breast cancer patients. Effective pain management post-surgery is critical for patient satisfaction, reducing complications, and facilitating quick recovery and hospital discharge. This study addresses the lack of patient-centered postoperative pain management guidelines for breast cancer patients.

Aim

The primary goal of this study was to develop tailored postoperative pain management guidelines for the local community in the United Arab Emirates, integrating these into a broader network of oncology facilities.

Methods and Materials

Employing a mixed-methods approach with a qualitative emphasis, the study gathered data from 10 female breast cancer patients (aged 39-65 years) with postoperative satisfaction surveys. Additionally, semi-structured interviews with six healthcare professionals involved in guideline development were conducted.

Results

A significant 90% of patients reported experiencing moderate-to-extreme pain post-surgery, indicating a need for improved pain management. Key factors identified included the need for enhanced nurse training and patient education on pain management preoperatively. The study team unanimously recognized the necessity for dedicated postoperative guidelines.

Conclusion

The study underscores the critical need for adequate postoperative pain management in breast cancer care. The findings advocate for creating multidisciplinary, evidence-based guidelines focused on patient-centered care. Furthermore, the study highlights the importance of international collaboration and continuous quality improvement measures, such as the Plan-Do-Study-Act (PDSA) cycle, for developing and refining these guidelines.

## Introduction

The critical importance of effective postoperative pain management in breast cancer surgery has been a significant focus of medical practice and research for over two decades, especially since 2000 when the Joint Commission on Accreditation of Healthcare Organizations mandated the assessment and management of pain [1]. Despite these mandates, challenges in managing certain types of postoperative pain adequately continue [2]. A study demonstrated that a substantial percentage of patients experienced post-surgical pain, with many reporting moderate-to-extreme pain levels persisting post-discharge [3].

Inadequate management of acute postoperative pain may lead to chronic persistent postsurgical pain, which is particularly prevalent in breast cancer surgery [4]. Suboptimal pain control can result in decreased functionality, impaired recovery, and diminished quality of life [5]. Therefore, developing and implementing comprehensive guidelines for postoperative pain management are crucial. Various organizations, including the American Society of Pain Management and the American Society of Anesthesiologists, have addressed this need by developing guidelines on perioperative and outpatient care [6,7].

The necessity for improved education in pain management among nursing professionals is evident. Research by Abdalrahim et al. emphasized the importance of established guidelines to enhance the quality of care, highlighting gaps in nurses' knowledge of postoperative pain management [8]. Wang and Tsai also found that the level of knowledge among nurses in this area was insufficient, as reflected in their performance on the Nurses’ Knowledge and Attitudes Survey [9].

Effective leadership plays a crucial role in healthcare, particularly in developing guidelines for postoperative pain management. Successful physician leaders are characterized by their competencies in trust, innovation, and effective communication [10]. Furthermore, Patel et al. identified essential leadership traits in surgery, such as technical expertise, motivation, and emotional intelligence [11].

Teamwork, under the guidance of effective leadership, is essential in healthcare. Leaders are responsible for fostering an environment of mutual respect and collaboration [12,13]. The relationship between empowering leadership, job satisfaction, and effective team self-leadership has been established [14]. Additionally, the connection between leadership and innovation in healthcare is vital. A large-scale study involving breast cancer care units demonstrated that leadership fosters team participation and innovation [15].

In response to these challenges, our center, a leading breast cancer treatment facility in the United Arab Emirates, focused on developing and implementing a comprehensive postoperative pain management strategy. Our methodology includes conducting patient surveys to assess satisfaction with current pain management strategies, thus emphasizing our commitment to patient-centered care. Moreover, networking with other oncology centers has been crucial in gathering insights for our guidelines, highlighting the significance of collaborative efforts in healthcare leadership.

## Materials and methods

This prospective study, proposed in an MSc leadership program, adopted a mixed-methods approach, combining qualitative and quantitative research techniques with a primary focus on qualitative data [16]. The study used a questionnaire informed by existing literature, featuring 10 questions adapted from the American Pain Society. These questions were designed to gauge patient satisfaction with postoperative pain management, employing a Likert scale for responses [17]. The questionnaire was offered to patients visiting the breast cancer clinic postoperatively and was translated into Arabic to accommodate the patient demographic. It included open-ended questions to allow for more detailed patient feedback.

In addition to the questionnaire, semi-structured interviews were conducted with six team members in an informal setting. Responses were recorded anonymously. The data from these interviews and the open-ended questionnaire responses were analyzed using thematic analysis methods [18]. Additionally, an electronic 360-degree feedback survey was distributed to four team members and the lead researcher's direct manager to assess the leadership role in the study.

The rationale behind employing both qualitative and quantitative methods was to leverage the strengths of each. Qualitative data, consisting of words and emotions, often informs subsequent interview questions. Quantitative data, represented numerically, offers statistically verifiable results, allowing for validation of the research quality. The qualitative approach facilitates an in-depth understanding of complex issues beyond the scope of quantitative analysis [19].

The study's participants included a purposeful sample of 10 female breast cancer patients aged 39 to 65 years who had scheduled postoperative appointments at the breast clinic. Similarly, a purposeful sample of 10 team members was selected, with six agreeing to participate. These team members represented diverse professional roles, including an anesthesiologist, a clinical pharmacist, a surgeon, a surgical resident, and a pain management nurse. The 360-degree feedback survey was circulated among four team members and the direct manager.

Data from the semi-structured interviews were manually analyzed, categorizing themes and subthemes [20]. The qualitative data were processed using Braun and Clarke's framework for thematic analysis. Quantitative data were analyzed using SPSS® Version 25 (IBM Corp., Armonk, NY) and Microsoft® Excel 2013 (Microsoft Corp., Redmond, WA), with frequencies and percentages being the primary statistical methods due to the small sample size.

Ethical considerations were meticulously addressed. Institutional and individual permissions were obtained, and participants provided written informed consent. The research adhered to ethical principles such as confidentiality, anonymity, and the “no-harm principle” outlined in the Belmont report [21]. To ensure rigor, unbiased questions were used in the interviews, and feedback regarding the researcher's leadership was ensured to have no impact on work relationships. A colleague independently reviewed the research paper to check for any biases, and data triangulation was employed using two different datasets.

## Results

**Figure 1:**
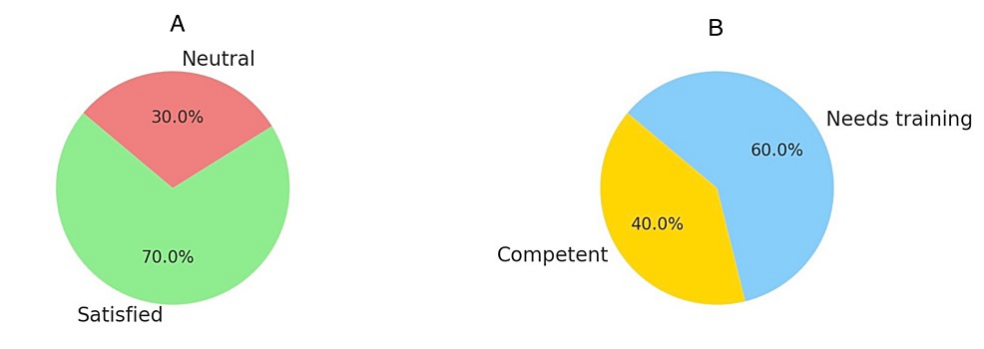
Nursing care satisfaction and competency (A) Patient’s satisfaction with the nursing care provided. (B) Patient’s perception of nursing care provided.

In this study, 90% (9 out of 10) experienced moderate-to-severe pain post-surgery, necessitating pain management. Nurses’ roles in postoperative care were crucial; 70% of patients were satisfied or very satisfied with their nursing care, 30% felt neutral, 40% viewed their nurse as competent, and 60% believed that further training was needed (Figures 1A, 1B).

**Figure 2:**
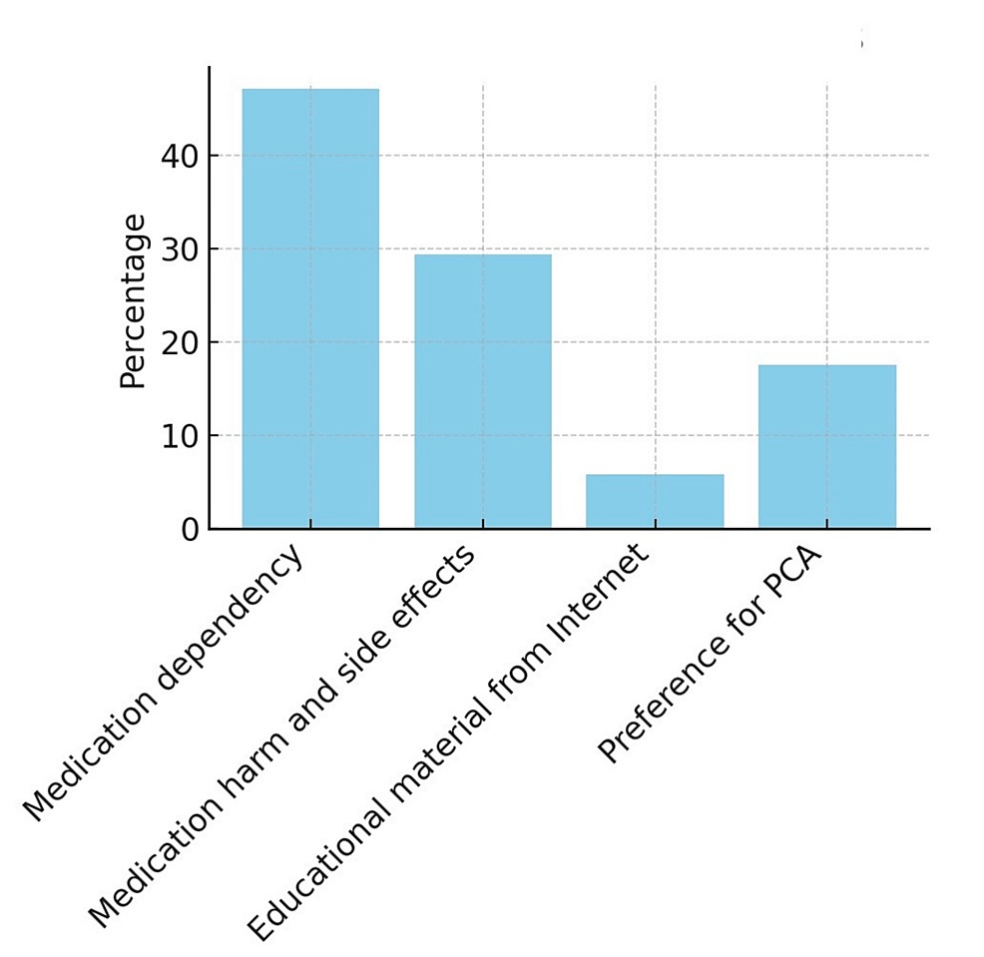
Patient concerns and preferences about postoperative pain management PCA, patient-controlled anesthesia

When asked about their concerns for postoperative pain management details, patients’ responses were as follows: the majority feared medication dependency (47.1%; eight patients), followed by fear of side effects (29.4%; five patients, 5.9% (one patient) reported that not enough education regarding pain management was provided and had to get information from the internet, and only 17.6% (three patients) preferred patient-controlled anesthesia for pain management (Figure 2).

In developing postoperative pain management guidelines, 66.7% of the team members advocated for the guidelines to be established at the local hospital level. In comparison, 33.3% considered the Department of Health as the appropriate body.

**Figure 3:**
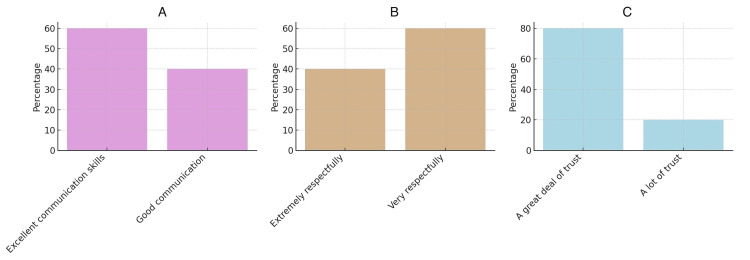
Communication skills, professionalism, and trust of the lead author (A). Communication skills. (B) Professionalism. (C) Trust.

The results of a 360-degree appraisal survey on the lead author’s leadership indicated that all respondents rated the lead author’s communication skills positively, ranging from good to excellent (Figure 3A). Moreover, 40% of the respondents perceived the lead author’s interactions as extremely respectful, underscoring their professionalism. In comparison, 80% of the team showed high trust in the lead author’s decision-making abilities, demonstrating strong teamwork and interpersonal skills (Figures 3B, 3C).

## Discussion

As observed in this study, the prevalence of postoperative pain is a critical concern, reinforcing the findings in the literature [22]. This study's higher pain incidence compared to other studies suggests the influence of cultural factors on pain perception, supporting the notion proposed by these studies that patient demographics and cultural backgrounds significantly impact pain experiences and management strategies [23,24].

Regarding nursing competency, the results were twofold. Some patients expressed dissatisfaction, resonating with a study highlighting a nurse training gap, particularly in pain management. In contrast, others acknowledged nurses' competencies. These diverse perceptions emphasize the need for continual professional development in nursing, as advocated by many studies, to enhance patient care and satisfaction [25].

Educational materials on postoperative pain management emerged as a significant area for improvement. Patients expressed concerns about understanding medication side effects and dependency risks, underscoring a gap in existing materials. This observation aligns with previous recommendations, emphasizing the importance of providing comprehensive, transparent, and accessible educational resources [26]. Such materials are essential for empowering patients to manage their pain following surgery.

The importance of robust, evidence-based guidelines for postoperative pain management is well recognized. Studies have emphasized the need for effective and safe post-surgical pain management strategies. Additionally, research has pointed out significant gaps in existing guidelines, highlighting the urgency for the development of comprehensive guidelines. It is also recommended that these guidelines be periodically reviewed and updated, incorporating the latest evidence and best practices to maintain their relevance and effectiveness in clinical settings [27].

The author's leadership role in this initiative was pivotal, with an emphasis on specific personality traits and skills. A 360-degree evaluation process played a key role in affirming this leadership, highlighting the importance of comprehensive feedback mechanisms in leadership development. Essential leadership qualities such as effective communication, professionalism, and teamwork were crucial in guiding the multidisciplinary team. These qualities are widely recognized as fundamental to successful leadership in healthcare settings [28]. The approach taken by the author aligns with contemporary views on innovation in leadership within healthcare, which stress the importance of dynamic and adaptable leadership qualities for implementing significant changes in healthcare settings. Recent studies and guidelines emphasize similar perspectives, underscoring the evolving nature of leadership roles in healthcare and the need for continuous development of these essential qualities [29].

In summary, this study identifies the critical areas in postoperative pain management and suggests potential avenues for improvement. It specifies the need for culturally sensitive, patient-centered approaches and the importance of continuous education and training for healthcare professionals. The study also highlights the crucial role of leadership in effecting change and driving improvements in patient care.

Limitations of the study

The survey may have had a limited number of respondents, and since they all come from the same department or share similar roles, this could skew the results. A diverse range of perspectives from different professional levels and departments would provide a more comprehensive understanding of the lead author's leadership qualities.

There is a risk of response bias, where respondents might provide socially desirable answers rather than honest feedback, especially if they believe their responses are not entirely anonymous [30]. This can lead to overestimating or underestimating the lead author's leadership abilities.

It is challenging to contextualize the results without a benchmark or comparative data from other leaders in similar roles. The absence of a control group or comparative analysis limits the ability to draw broad conclusions about the lead author's leadership effectiveness.

## Conclusions

This study highlights the critical need for enhanced postoperative pain management and nursing competency in healthcare settings. The high incidence of postoperative pain, as reported by the majority of patients, points to the necessity for culturally sensitive and patient-centered pain management approaches. Additionally, the varied perceptions of nursing competency suggest a need for ongoing professional development and training in pain management for nursing staff. The study also emphasizes the importance of providing patients with comprehensive, clear, and accessible educational materials on pain management, medication side effects, and dependency risks. The development and implementation of evidence-based postoperative pain management guidelines are paramount, as is the role of effective leadership in driving these improvements. Overall, this study provides valuable insights into improving postoperative care and underscores the importance of multifaceted approaches in enhancing patient satisfaction and outcomes in healthcare settings.
